# Variable cost of a Greek ICU

**DOI:** 10.1186/cc12423

**Published:** 2013-03-19

**Authors:** E Tsigou, D Karabatsou, E Boutzouka, V Psallida, G Baltopoulos, M Tsironi

**Affiliations:** 1 A. Anargiroi Hospital, Athens, Greece; 2 University of Peloponnisos, Sparti, Greece

## Introduction

The ICU consumes almost 20% of total hospital resources, despite accounting for less than 10% of hospital beds. Medical cost can be divided into a fixed part, consisting of personnel and accommodation expenses, and a variable part, determined by the needs of each patient (medications, consumables, examinations). In order to rationalize medical cost, estimation of expenses must be real and individualized. The aim of this study was to assess the variable cost of critically ill patients in a new, seven-bed, adult, general ICU, using bottom-up costing methodology.

## Methods

All 138 patients who were treated during 2011, and stayed for at least 24 hours, were included in the study. Data were retrospectively collected from patient records and included demographics, cause of admission, APACHE II score at admission, length of stay (LOS) and outcome. Cost was recorded for every day and for every patient, based on a hospital-specific cost catalogue and on national agreements. Data are presented as mean ± SEM. Analysis of data was carried out using Graph Pad Prism 5.0., applying Student's *t *test.

## Results

The age of participants (84 men and 54 women, 71 medical and 67 surgical) was 68.75 ± 1.18, APACHE II score was 18.64 ± 0.61, LOS was 18.46 ± 2.54, and mortality was 19%. The majority of the patients (61%) were mechanically ventilated. The total days of ICU stay were 2,548 and the total variable cost was €1,460,465 (€573.18 per patient and per days). Cost per patient was subdivided as follows: medication (including drugs, fluids, blood products, nutrition): 56.49%, examinations (including laboratory, microbiological assays and diagnostic procedures): 22.23%, consumables: 21.26%. As for medication cost, the largest part was comprised of antibiotics (45.69%), followed by blood products (17.61%) and cardiovascular drugs (12.39%). Costs for medical patients were significantly higher than those for surgical patients (*P *0.0001).

## Conclusion

The total average cost per patient and per days was found to be € 573.18. Medication expenditure was responsible for the highest variable cost of intensive care treatment. Among drugs, antibiotics accounted for the largest part. A medical cause of admission was correlated with higher costs.

## References

[B1] McLaughlin AM Intensive Care Med 2009 35 2135 2140 10.1007/s00134-009-1622-1 19756509

[B2] Rossi C Intensive Care Med 2006 32 545 552 10.1007/s00134-006-0080-2 16501946

